# A model of quiescent tumour microregions for evaluating multicellular resistance to chemotherapeutic drugs

**DOI:** 10.1038/sj.bjc.6602710

**Published:** 2005-07-19

**Authors:** H R Mellor, D J P Ferguson, R Callaghan

**Affiliations:** 1 Oxford Drug Resistance Group, Nuffield Department of Clinical Laboratory Sciences, John Radcliffe Hospital, University of Oxford, Oxford OX3 9DU, UK; 2 Ultrastructural Morphology Group, Nuffield Department of Clinical Laboratory Sciences, John Radcliffe Hospital, University of Oxford, Oxford OX3 9DU, UK

**Keywords:** quiescent, tumour, spheroid, microenvironment, chemotherapy, multicellular resistance

## Abstract

The quiescent cell population of tumours poses a barrier to the success of many cancer therapies. Most chemotherapeutic drugs target proliferating cells, but the growth fraction of many tumours is low. Based on the multicellular tumour spheroid model, a system was developed using human colon adenocarcinoma (DLD-1) cells to mimic the microenvironment of quiescent microregions of solid tumours. The quiescent tumour spheroids (TS^Q^) showed decreased expression of the proliferation marker Ki-67 and increased expression of the quiescence marker p27^kip1^ compared to proliferating spheroids (TS^P^). The quiescent status of the TS^Q^ was confirmed by long-term growth assessment. The quiescence was completely reversible demonstrating that the TS^Q^ retained the ability to proliferate and morphological assessment by light microscopy confirmed the absence of significant apoptosis. When the efficacy of widely used chemotherapeutic drugs was determined, vinblastine, doxorubicin, cisplatin and 5-fluorouracil (5-FU) all produced significant cell death in the TS^P^. However, while still effective, the potencies of doxorubicin and cisplatin were significantly reduced in TS^Q^. In contrast, 5-FU and vinblastine did not produce cell death in the TS^Q^. In summary, TS^Q^ show considerable resistance to a panel of established chemotherapeutic agents and represent a useful model for evaluating the efficacy of drugs and other cancer therapies in quiescent tumours.

In many tumours, the actively dividing cells account for only a small proportion of the total, with the remainder of the cells being in a quiescent state (Q-cells) (Jackson, 1989). For particularly slow growing tumours (e.g. prostatic), a mean growth fraction of <3% has been reported (Gallee *et al*, 1989; Berges *et al*, 1995). The quiescent cells within the tumour are viable but in a reversible state of growth arrest (Desoize and Jardillier, 2000). The mechanism behind the development of an intratumoural population of Q-cells remains unclear, but it may be a consequence of multiple microenvironmental factors, which result from the abnormal/inadequate vasculature often found within solid tumours (Brown and Giaccia, 1998). For example, hypoxia has been shown to evoke cell cycle arrest via the cyclin-dependent kinase (CDK) inhibitor p27^kip1^ (Gardner *et al*, 2001).

The relative expression levels of cellular proliferation (e.g. Ki-67) and quiescence (e.g. p27^kip1^) markers are valuable in determining the aggressiveness of tumours and therefore for predicting prognosis. A high proportion of Ki-67-positive cells in a tumour is indicative of a poor prognosis (Scholzen and Gerdes, 2000). Similarly, patients with tumours expressing low levels of p27^kip1^ have a poorer prognosis than those whose tumours have high p27^kip1^ expression. Such correlations have been shown for many different human cancers (Blain *et al*, 2003). However, aggressive tumours with a high proportion of Ki-67-positive cells, despite imparting a poorer prognosis upon the patient, usually respond well to chemotherapy (Kamoi *et al*, 2001; Petit *et al*, 2004).

The selectivity of chemotherapeutic agents for killing proliferating cells (P-cells) over Q-cells has been demonstrated for many classes of drugs in cell monolayers (Valeriote and van Putten, 1975; Siu *et al*, 1999) and in the solid tumours of patients (Remvikos *et al*, 1993; Hietanen *et al*, 1995). In addition to their inherent chemoresistance, the inadequate vascularisation in tumour regions occupied by Q-cells leads to low local concentrations of drugs. As a consequence, the large Q-cell population of many human carcinomas (e.g. colon carcinoma, non-small-cell lung carcinoma) contributes to these tumours being refractory to many chemotherapy regimens (Jackson, 1989).

The insensitivity of Q-cells to chemotherapeutic drugs also contributes to the additional problem of regrowth resistance, through recruitment of the surviving Q-cells to the P-fraction after drug treatment (Brown and Giaccia, 1998; Siemann, 1998; Desoize and Jardillier, 2000). The low growth fraction of many tumours leads to the requirement for multiple repeated drug treatments over a prolonged time period (Shah and Schwartz, 2000). Finally, certain chemotherapeutic drugs can themselves induce quiescence in tumour cells (Carroll *et al*, 2003), rendering these cells refractory to the effects of further drug intervention.

Therefore, an important question is how to treat those more indolent solid tumours that are chemoresistant due to a Q-cell population? Even for emerging cancer treatments such as gene therapy, tumour Q-cells pose a significant barrier to successful treatment. For example, the use of many retroviral vectors is significantly hampered by the dependence on host cell proliferation for viral replication (Patton *et al*, 2004). It is of paramount importance that we gain a greater insight into the efficacy of currently available therapies for killing tumour Q-cells and to develop novel drugs or therapeutic approaches that are more selective for this cell population.

In order to screen and evaluate chemotherapies and other therapeutic approaches, a suitable model system is required that is representative of the quiescent regions of solid tumours. Adapted monolayer cultures have been previously used to model populations of quiescent tumour cells (Phillips and Clayton, 1997; Horiatis *et al*, 2004). However, monolayers do not pose the barrier to drug penetration or provide many of the microenvironmental influences found in solid tumours and 3D cultures (Desoize and Jardillier, 2000). The multicellular tumour spheroid (MCTS) model (Inch *et al*, 1970; Sutherland *et al*, 1971; Hamilton, 1998) involves the culturing of cancer cell lines as 3D structures. In the absence of a suitable tissue culture system, we developed and evaluated a TS model of quiescent tumour microregions (TS^Q^) and compared them to proliferating spheroids (TS^P^). We chose to establish the TS using a DLD-1 human colon adenocarcinoma cell line as the mean doubling time of cancer of the colon is 90 weeks (Slingerland and Tannock, 1998) and this disease is synonymous with a poor chemotherapeutic response. To evaluate the usefulness of this new system, immunohistochemical detection of the proliferation marker Ki-67 and the quiescence marker p27^kip1^ was performed to assess the localisation of P- and Q-cells within the TS. The long-term growth characteristics of both types of TS were measured and the viability of the TS^Q^ after long-term quiescence was studied. In order to compare drug resistance profiles in the TS^P^ and TS^Q^, a novel method for quantifying cells within the spheroid mass was developed and utilised to test a series of commonly used chemotherapeutic agents. We chose to study the long-term response to drug treatment as discrepancies between short- and long-term responses have previously been observed when comparing the effects of drugs in P- and Q-cells (Siu *et al*, 1999).

## MATERIALS AND METHODS

### Materials

RPMI-1640 medium (with Glutamax I and 25 mM HEPES), foetal bovine serum (heat inactivated), penicillin/streptomycin solution and trypsin-EDTA were purchased from Life Technologies Ltd, UK. Methylene blue and fatty acid-free BSA were purchased from Sigma, UK. For the immunohistochemistry, Ki-67 and p27^kip1^ mouse anti-human monoclonal antibodies were obtained from DakoCytomation, UK. Mach-2 polymer-HRP conjugate and peroxidazed 1 were from BioCarta, UK. Haematoxylin and aquamount were obtained from BDH Laboratory Supplies, UK. Vinblastine sulphate, doxorubicin hydrochloride and 5-fluorouracil (5-FU) were purchased from Sigma. Cisplatin was obtained from Professor Trevor W Hambley (University of Sydney, Australia).

### Cell lines, culture and TS production

DLD-1 human colon adenocarcinoma cells were obtained from Dr Roger Phillips (University of Bradford, UK) and were grown as monolayers in RPMI-1640 medium (with Glutamax I and 25 mM HEPES) supplemented with 10% (v v^−1^) fetal bovine serum and penicillin (100 IU ml^−1^)/streptomycin (100 mg ml^−1^). For most experiments, TS of DLD-1 cells were grown in spinner flasks (Techne, UK). A mother dish was prepared by coating the base of a T75 cm^2^ cell culture flask with 20 ml of 0.75% (w v^−1^) agarose prepared in medium without serum. Exponentially growing DLD-1 cells were added in 20 ml of medium at a density of 1.5 × 10^6^ cells ml^−1^ and the flask was left stationary for 24 h (37°C, 5% CO_2_). Any large-cell aggregates were removed by gravity sedimentation and the remaining small aggregates were transferred to a 500 ml spinner culture flask and made to a total volume of 100 ml of serum containing medium. The spinner flask was then placed on a stirrer (Techne MCS-1045) at 37°C, 5% CO_2_ for an initial period of 3 days to allow the TS^P^ to form. To generate TS^Q^, the TS^P^ were washed with PBS and fresh medium containing 0.1% fatty acid-free BSA instead of 10% FCS was added. For continuous culturing of TS^P^ and TS^Q^, the appropriate medium was refreshed (50 ml) every other day.

### TS growth curves

In order to monitor growth of TS^Q^ and TS^P^, TS were removed from the spinner flask to a Petri dish in a small volume of medium. The diameter of TS (*n*≃20) was measured daily, at the same time of day, and after measurements had been taken, the TS were replaced in the spinner flask. A graduated calibrated microscope eyepiece graticule (Pyser-SGI, UK) was used to measure TS diameter. TS diameter was converted into micrometers (*μ*m), which allowed calculation of TS volume (*μ*m^3^). Growth curves for TS represent the mean±s.e.m. of three independent experiments.

### Routine histological procedures

TS were harvested, washed in PBS and fixed in neutral-buffered formalin (pH 7.0). The TS were transferred to small plastic casting moulds for embedding, allowed to settle and the formalin removed. The moulds were filled with melted 2% (w v^−1^) agarose in 4% (v v^−1^) formaldehyde. The agarose was allowed to set on ice before being removed from the mould and placed in a tissue cassette, which was routinely processed (Histopathology Department, John Radcliffe Hospital, Oxford, UK). The processed, agarose-embedded, TS were embedded in paraffin wax and 5 *μ*m sections were cut.

### Proliferation status of TS^P^ and TS^Q^: Ki-67 and p27^kip1^

TS sections (day 12) were completely dewaxed and rehydrated with PBS, placed in 50 mM Tris/200 mM EDTA buffer, pH 9.0, and antigen retrieval was carried out in a Decloaking Chamber Pro (Biocarta, UK) at 120°C for 30 s. Sections were washed with PBS and incubated in a humidified chamber (25°C) with peroxidazed for 10 min to inhibit endogenous peroxidase activity. After washing the sections with PBS, the primary monoclonal antibody (mouse anti-human), Ki-67 (1 : 100 in PBS) or p27^kip1^ (1:50 in PBS), was added for 45 min at RT. Mach-2 goat-anti-mouse HRP conjugate was added for 45 min and detection was achieved using DAB substrate chromagen. Sections were counterstained with haematoxylin and mounted with aquamount.

### Calibration of cell number in TS by methylene blue staining

Freshly trypsinised DLD-1 cells taken from exponentially growing cultures were overlaid on solid agarose base-coats at densities ranging from 1 × 10^3^ to 3 × 10^4^ cells in 100 *μ*l medium. The cells were kept stationary for 24 h (37°, 5% CO_2_) after which the plates were transferred to a Titramax 100 (Heidolph Instruments, Germany) and shaken at 300 r.p.m. for a further 24 h (37°C, 5% CO_2_) to allow TS to form. The TS were moved to a new ‘uncoated’ well in a 48-well plate, 500 *μ*l of fresh medium was added and TS were allowed to adhere for 48 h (37°C, 5% CO_2_). After this time, the medium was aspirated and replaced with 200 *μ*l of 5 g l^−1^ methylene blue in methanol, for 30 min, to fix and stain the cells. The wells were washed five times with tap water to remove excess dye and the plates were allowed to dry overnight (25°C). The stained TS^P^ were solubilised with 200 *μ*l of 2% SDS by shaking on a Titramax 100 at 300 r.p.m. for 24 h (25°C). A 100 *μ*l aliquot was taken from each well and placed in the well of a 96-well plate and the absorbance was measured at 650 nm in a Spectra Max 250 microplate reader (Molecular Devices, UK).

### Drug cytotoxicity measurements in TS

Proliferating tumour spheroids were grown as described above and on day 6 (when they have no inherent Q-cells) were transferred in 100 *μ*l of fresh medium to 96-well tissue culture plates, which had previously been given a 100 *μ*l base-coat of 0.75% (w v^−1^) agarose (prepared in RPMI-1640 medium). Drugs were prepared in medium at twice the desired concentration to give a final concentration range of 1 nM to 316 *μ*M (up to 1 mM for 5-FU) and 100 *μ*l aliquots were added to each well. After the 16 h incubation, the TS^P^ were moved to a new ‘uncoated’ well in a 48-well plate and 500 *μ*l of fresh medium added. The TS^P^ were incubated for a further 6-day recovery period. After this time, the methylene blue assay was performed as described above.

The TS^Q^ were generated from TS^P^ at 6 days growth by switching the medium to serum-free as described above and all subsequent culturing of TS^Q^ was performed in this medium. The drug exposure and recovery protocol used for TS^P^ was applied to the TS^Q^; however, a slightly modified staining procedure was applied due to nonadherent nature of the TS^Q^. After the 6-day recovery period, TS^Q^ were transferred to the wells of a round-bottomed 96-well plate, the medium was aspirated and 200 *μ*l of 5 g l^−1^ methylene blue in methanol was added to each well for 30 min. The dye was aspirated, and the fixed spheroids were washed three times with 250 *μ*l of tap water. The solubilisation procedure and absorbance measurements were performed as above.

### Morphological evaluation of TS

For morphological examination and comparison of the different TS models, TS^Q^ at the end of the growth study (i.e. day 25) and TS^P^ of equivalent size (approximately day 8) were used. For drug-treated samples, day 6 TS^P^ or TS^Q^ were transferred to agarose-coated 96-well plates and exposed to vinblastine (3 *μ*M), doxorubicin (3 *μ*M) or cisplatin (30 *μ*M) for 48 h. This period of exposure to the above concentrations of these drugs had previously been shown to result in morphologically observable cytotoxicity in proliferating TS (Hall *et al*, 2004). TS were washed in PBS and fixed in 4% glutaraldehyde in 0.1 M phosphate buffer. Samples were postfixed in osmium tetroxide, dehydrated in ethanol, treated with propylene oxide and embedded in Spurr's epoxy resin. Sections were cut at 1 *μ*m thick and were stained with Azure A. Morphological features were identified by light microscopy. The number of apoptotic and mitotic cells per spheroid section was counted.

### Data analysis

Data analysis was performed using GraphPad Prism™ software. Spheroid growth data were fitted with an exponential growth curve using the equation *Y*=*A*e^*KX*^, where *Y* is the spheroid volume (*μ*m^3^) and *X* is time (days). *Y* starts at *A* and increases geometrically with a doubling time equal to 0.6932/*K*. The doubling times obtained from the curves were compared based on 95% confidence intervals using one-way ANOVA and Tukey's post-test. Drug cytotoxicity in TS was quantified using nonlinear regression as described in (Hall *et al*, 2004).

## RESULTS

### TS markers of proliferation and quiescence

Serum withdrawal is a well-established method of inducing quiescence in cultured cell lines and has previously been used to induce quiescence in DLD-1 cells (Shida *et al*, 2003). However, as far as we are aware, this approach has not previously been applied to TS. To characterise the proliferative changes occurring within the TS tissue, spheroids were immunostained for the proliferation marker Ki-67 and the quiescence marker p27^kip1^.

**Figure 1:**
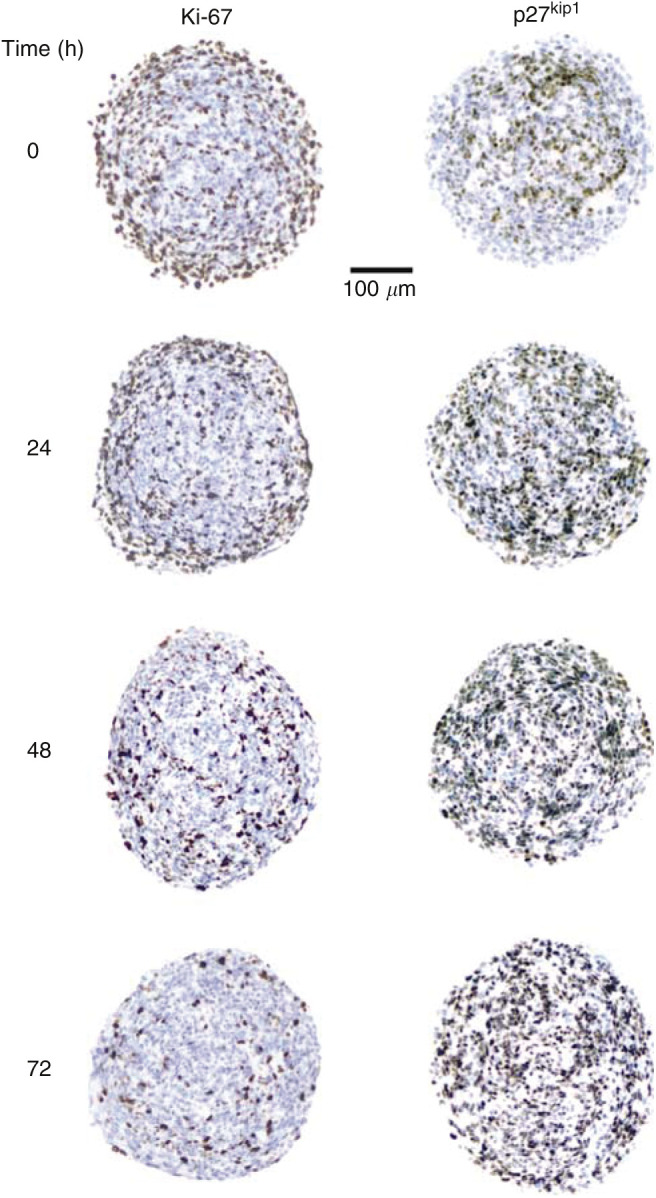
Time course of serum withdrawal on expression of Ki-67 and p27^kip1^ in TS. The TS growth medium was replaced with fresh medium containing 0.1% fatty acid-free BSA instead of 10% FCS. TS were harvested at various times over a period of 72 h and were fixed in formalin, routinely processed and embedded in paraffin wax. Immunohistochemistry was performed on sections (5 *μ*m) to detect the proliferation marker Ki-67 and the quiescence marker p27^kip1^. Sections were counterstained with haematoxylin and mounted with aquamount. The scale bar corresponds to 100 *μ*m.

Initial investigations examined the effect of serum withdrawal on Ki-67 expression in the TS. Prior to serum withdrawal (*t*=0 h), a large proportion of the cells within the tissue were Ki-67-positive, particularly those at the periphery, with a small nonproliferating population located at the centre of the TS (Figure 1), an expected feature of TS of intermediate size (day 12). After 24 h in serum-free medium, Ki-67 expression was relatively unchanged; however, by 48 h a dramatic decrease was evident. Moreover, the location of the few Ki-67-positive cells was no longer biased to the periphery. A further decrease in expression was seen after 72 h, by which point only a residual number of cells appeared to be proliferating (Figure 1).

In addition to the dramatic decrease observed in the number of Ki-67-positive cells within the TS, a concurrent increase in p27^kip1^-positive cells was seen during the 72 h period of serum starvation. Initially (*t*=0 h), the central region of the TS contained a number of p27^kip1^-positive cells (Figure 1), consistent with the absence of Ki-67 staining in this region. However, in contrast to the dramatic decrease in KI-67 that occurred between 24 and 48 h, p27^kip1^ expression increased gradually over the 72 h period. At 72 h, almost every cell was found to be p27^kip1^-positive, consistent with the almost complete absence of Ki-67 expression at this time (Figure 1). The change in Ki-67/p27^kip1^ expression was reversible, over a 72 h period, upon readministration of serum-containing medium (data not shown).

### Growth curves of TS^Q^ and TS^P^

**Figure 2:**
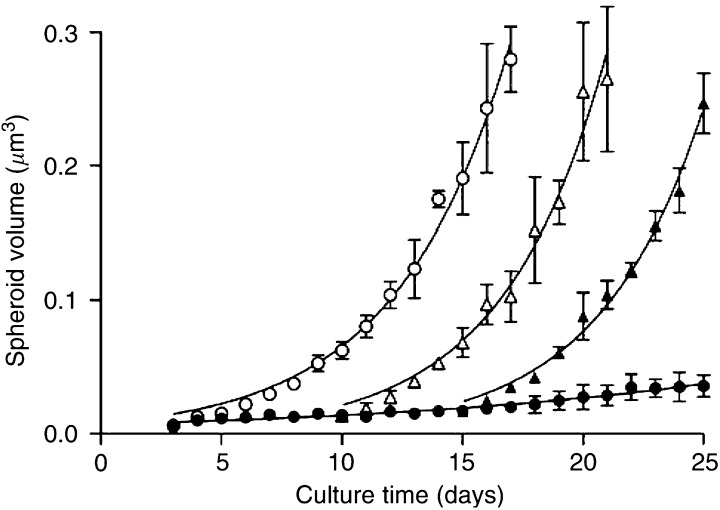
Growth curves for TS and reversibility of long-term quiescence. To generate TS^Q^ (black circles), 3 days after seeding the spinner flask (day 3) TS^P^ (open circles) were washed with PBS and fresh medium was added containing 0.1% fatty acid-free BSA instead of 10% FCS. To assess the reversibility of the growth arrest evoked by serum withdrawal, the TS^Q^ growth medium was switched back to serum-containing day 10 (open triangles) and again on day 15 (closed triangles). For continuous culturing of TS^P^ and TS^Q^, the appropriate medium was refreshed (50 ml) every other day. In order to monitor growth of TS^Q^ and TS^P^, TS were removed from the spinner flask to a Petri dish in a small volume of medium. The diameter of TS (*n*≃20) was measured daily and was used to calculate TS volume (*μ*m^3^). Growth curves represent the mean±s.e.m. of three independent experiments.

The growth rates of the quiescent spheroids (TS^Q^) were compared with those of actively proliferating spheroids (TS^P^) to further characterise the model. As shown in Figure 2, the effect of culturing TS in serum-free medium was dramatic. From day 3, when the medium was changed, the curves for the TS^P^ and TS^Q^ diverged. The TS^P^ displayed sustained, exponential growth, which continued up to day 17 when the spheroid volume was ∼0.28 *μ*m^3^ (*d*≃800 *μ*m). At this point, the spheroids collapsed, most likely due to the large area of central necrosis that the TS^P^ developed by this late stage. In comparison, the TS^Q^ showed minimal growth on a day-to-day basis, although an increase in spheroidal volume (0.007±0.002 to 0.036±0.008 *μ*m^3^) was evident over the 25-day observation period (Figure 2). This small volume increase indicates that a small proportion of cells were capable of proliferation even in the absence of serum. The growth differences between the two types of TS is highlighted by the fact that between day 3 and day 17, the TS^P^ demonstrated a 48-fold increase in volume compared to only a 2.5-fold increase for the TS^Q^.

The aim of developing the TS^Q^ model was to enable examination of the sensitivity of quiescent tumour microregions to chemotherapeutic agents. In order to achieve this, it was important to establish that the serum-free medium used to generate the model did not have any deleterious effects on the viability of cells within the TS^Q^, other than to restrict cell division. Therefore, the reversibility of the serum withdrawal was checked by replacing the TS^Q^ growth medium with serum-replete. This was carried out on day 10 and again on day 15 and the ability of the TS^Q^ to proliferate was assessed. The addition of serum to the culture medium rapidly returned the TS^Q^ to a proliferative state (Figure 2). This was found to be the case for TS^Q^ switched at day 10 (open triangles) or even later, at day 15 (closed triangles). Once proliferation had been initiated, these TS followed the same pattern of growth as TS^P^. No statistically significant difference was observed in the rate of proliferation between the TS^P^ cultured in serum throughout from those that had been switched from a quiescent to a proliferative state. A significant difference (*P*<0.01) was found between the doubling time of the TS^Q^ (11.7±2.1 days) and each of the TS^P^ groups (3.0±0.1 days).

### Efficacy of chemotherapeutic drugs in TS

**Figure 3:**
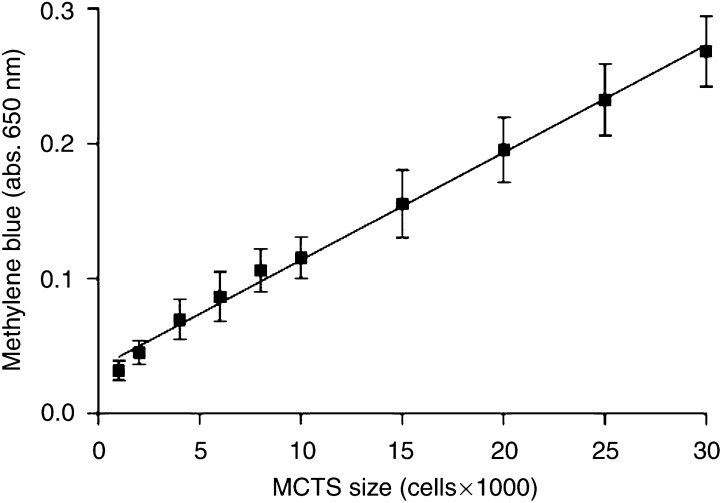
Standard curve for methylene blue staining against TS cell number. To ensure methylene blue staining was representative of the total number of cells present in a 3D TS, the assay was performed on TS of defined cell number from 1 × 10^3^ to 3 × 10^4^ cells. Staining, solubilisation and absorbance measurements were carried out and the standard curve of absorbance against TS cell number was plotted.

To assess the cytotoxicity of various chemotherapeutic agents in TS^Q^ and TS^P^, we developed a modified version of the methylene blue TS outgrowth assay. The original assay was unsuitable for the nonproliferating TS^Q^ due to the lack of cellular outgrowth. Consequently, the assay was modified to quantify the amount of dye associated with the TS after staining. This provided a reliable, quantitative measure of total TS cell number. The linearity of the assay was checked by generating TS with a wide range of sizes (1 × 10^3^ to 3 × 10^4^ cells). A linear relationship was found between cell number and methylene blue absorbance for TS over the range of sizes tested (Figure 3), and therefore the assay was a way of assessing drug cytotoxicity in the TS.

TS of ∼1 × 10^4^ cells (day 6 of spinner flask culture) were chosen for the cytotoxicity assays as these TS consisted entirely of proliferating cells and were within the linear range of sizes defined by the standard curve (Figure 3). This allowed for substantial drug-induced cell loss to be measured. A 16 h drug exposure time was employed for the cytotoxicity assays, followed by a 6-day ‘recovery’ period designed to allow any cytotoxic effects imparted by the drugs to be fully realised in the TS^P^ and TS^Q^.

The drugs chosen were selected on the basis of their different mechanisms of action and due to their established and widespread use in the clinic. For all of the chemotherapeutic agents tested, the potency in the TS^Q^ was significantly lower than that observed in the TS^P^ (Table 1). All of the drugs demonstrated considerable cytotoxicity in the TS^P^, vinblastine and doxorubicin being equipotent with an IC_50_ of ∼1 *μ*M. Cisplatin (IC_50_ 3.3±1.2 *μ*M) and 5-FU (IC_50_ 25±8 *μ*M) also produced significant inhibition of TS^P^ growth, although their potencies were reduced. The extent of cell kill at the maximum drug concentration was high for all of the drugs (>85% see Table 1), demonstrating that the majority of cells within the TS^P^ were drug-sensitive. Despite the high concentrations of drugs used (up to 316 *μ*M except 5-FU (1 mM)), the TS^Q^ were mostly insensitive to the effects of two of the agents, the vinca alkaloid vinblastine and the antimetabolite 5-FU. The mean extent of cell kill was 34±12 and 11±11% for the respective compounds. As a result of the lack of cytotoxicity, no meaningful IC_50_ was achievable for these compounds (Table 1).

In contrast, both doxorubicin and cisplatin displayed considerable cytotoxicity in the TS^Q^. The potency of doxorubicin in the TS^Q^ (13±5 *μ*M) was 16-fold lower than that displayed in the TS^P^ and the potency of cisplatin in the TS^Q^ (84±30 *μ*M) was 38-fold lower than that displayed in the TS^P^. As observed in the TS^P^, the majority of the TS^Q^ cells were sensitive to the effects of doxorubicin and cisplatin with a maximum cell kill of 81±10 and 80±12%, respectively.

### Morphological analysis of TS

**Figure 4:**
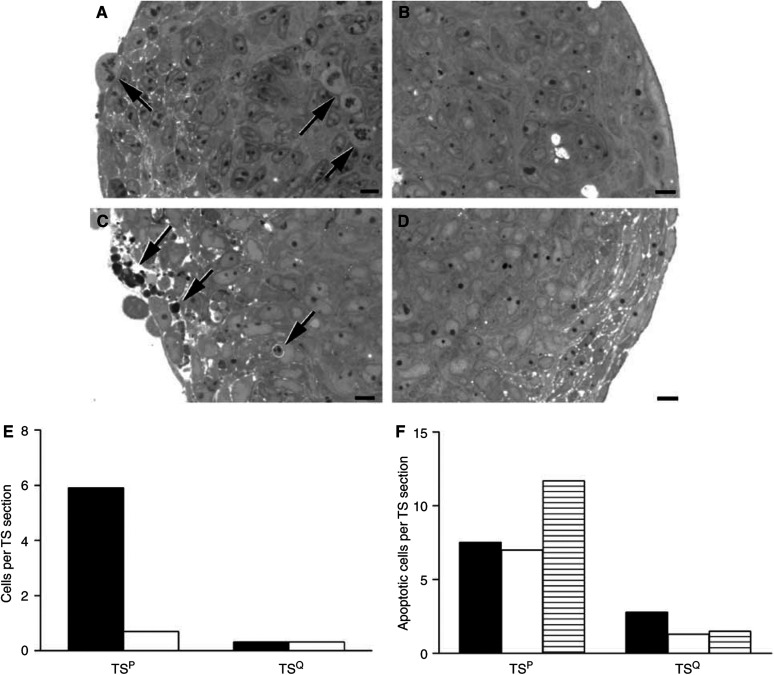
Effects of chemotherapeutic drug treatment on the morphology of TS. TS were fixed in 4% glutaraldehyde in 0.1 M phosphate buffer, postfixed in osmium tetroxide, dehydrated in ethanol, treated with propylene oxide and embedded in Spurr's epoxy resin. Sections were cut at 1 *μ*m thick and stained with Azure A. Images are representative of the typical appearance. The scale bar represents 10 *μ*m. (**A**) Untreated TS^P^ showing a number of mitotic cells (arrows). (**B**) Untreated TS^Q^ consisting of interphase cells with very few mitotic or apoptotic cells. (**C**) TS^P^ treated with cisplatin (3 *μ*M) for 48 h showing a number of apoptotic bodies (arrows). (**D**) TS^Q^ treated with cisplatin (3 *μ*M) for 48 h showing little evidence of apoptosis. (**E**) The number of mitotic cells (black bars) and apoptotic bodies (white bars) expressed as a mean per spheroid section for TS^P^ and TS^Q^. (**F**) The number of apoptotic bodies in TS^P^ and TS^Q^ treated with doxorubicin (black bars), vinblastine (white bars) and cisplatin (lined bars) expressed as a mean per spheroid section.

Morphological findings in the TS^P^ and TS^Q^ supported the previous experimental observations. A number of mitotic cells (mean 5.9 per TS, Figure 4E) were observed in the outer regions of the TS^P^ (Figure 4A), but few (mean 0.33 per TS, Figure 4E) were observed in the TS^Q^ (Figure 4B). This is consistent with the change in expression of proliferation markers (Figure 1) and the difference in growth of the TS^Q^ compared with the TS^P^ (Figure 2). Long-term culturing of TS^Q^ in 0.1% BSA (day 25) did not lead to an increase in the number of apoptotic bodies (Figure 4B) (mean 0.33 per TS section, Figure 4E) compared to the TS^P^ (mean 0.71 per TS section, Figure 4E). This is consistent with growth arrest due to a decrease in mitosis and is not associated with increased apoptosis. It is also consistent with the finding that the quiescent status of the TS^Q^ is fully reversible to TS^P^ with no decrease in the rate of growth (Figure 2). There appeared to be some deposition of glycogen in the TS^Q^, previously shown to be associated with decreased cell division rates in human colon carcinoma cell lines (Rousset *et al*, 1980), and therefore consistent with long-term culturing of DLD-1 TS^Q^. Cisplatin-treated TS^P^ contained a number of apoptotic bodies (Figure 4C). Although there were some apoptotic bodies in the cisplatin-treated TS^Q^ (Figure 4F), there were often none in a particular field (Figure 4D). For all of the chemotherapeutic drugs, a reduced level of apoptosis was observed in the TS^Q^ compared to the TS^P^ (Figure 4F). This is consistent with the cytotoxicity assays where a decrease in the potencies of all of the drugs tested was observed in the TS^Q^ relative to the TS^P^ (Table 1).

## DISCUSSION

In general, slowly growing tumours tend to be less drug-sensitive than rapidly growing tumours. Of the various cytokinetic factors that contribute to tumours having slow growth rates (e.g. long cycle times, high cell loss, Q-cells) (Riedel, 2002), modelling studies suggest that the presence of a large Q-cell compartment has the most serious implications for chemotherapy (Jackson, 1989). Most proliferation models are based on cell monolayers (Horiatis *et al*, 2004), but it is well established that for many chemotherapeutic drugs, a solid tissue environment affords an increased level of drug resistance (Desoize and Jardillier, 2000; Hall *et al*, 2004) called multicellular resistance. It is therefore of paramount importance in the evaluation of current and future therapies that a suitable system is developed to better represent the multicellular quiescent microregions in solid tumours.

Serum withdrawal is an established way of evoking growth arrest in proliferating cells, and although this approach has been used for DLD-1 cells (Shida *et al*, 2003), it has not previously been applied to TS. Ki-67 is a nuclear protein present during all active phases of the cell cycle in both normal and cancerous cells. It is expressed in P-cells but not Q-cells and is therefore a useful marker for cellular proliferation (Endl and Gerdes, 2000; Scholzen and Gerdes, 2000). We observed that after 72 h in serum-free medium, very few cells within the TS expressed the protein and were therefore no longer proliferating. To observe the switch from proliferating to quiescent cells within the TS, immunostaining for p27^kip1^ was performed. The levels of this protein are increased in quiescent cells (Lloyd *et al*, 1999) and constitutive expression in cultured cells leads to arrest in G_1_ phase of the cell cycle (Toyoshima and Hunter, 1994). In response to serum withdrawal, we observed a dramatic increased in expression of p27^kip1^ that was seen throughout the TS by 72 h. This was consistent with the Ki-67 staining results and demonstrated that the cells had entered a quiescent state.

In addition to displaying markers of cellular quiescence, the TS^Q^ showed minimal signs of growth over the 25-day measurement period. However, the spheroids retained the capacity for proliferation as was clear from the dramatic increase in TS size when they were exposed to serum-containing medium. This proliferative potential retained by quiescent cells forms the basis for the regrowth resistance that arises after chemotherapy or ionising radiation treatments when these insensitive cells repopulate the tumour (Desoize and Jardillier, 2000). It was clear from both the proliferative response of the TS^Q^ and the microscopy data that there were no deleterious effects of serum withdrawal on the TS^Q^. This was an important factor in the use of the system for the assessment of drug-induced cytotoxicity.

In order to test our model, we exposed the TS^Q^ and TS^P^ to anticancer drugs with different mechanisms of action. All of the drugs were less effective in the TS^Q^ relative to the TS^P^, highlighting the inherent drug resistance of these quiescent microregions. The two phase-specific agents we studied, 5-FU (S phase) and vinblastine (M phase), were both ineffective in the multicellular environment of the TS^Q^. The mechanism of action of the uracil analogue 5-FU is through incorporation of its metabolites into RNA and DNA and through thymidylate synthase inhibition (Longley *et al*, 2003), which is required for DNA replication and repair. The inability of 5-FU to exert any cytotxicity in the TS^Q^ is consistent with the lack of replicating cells compared to the TS^P^. Vinblastine, along with vincristine, is a naturally occurring member of vinca alkaloid class of anticancer drugs. It is an antimitotic agent that blocks cells in metaphase through suppression of microtubule dynamics (Jordan and Wilson, 2004). The selectivity of the drug for cells undergoing mitosis explains why the nondividing TS^Q^ were unresponsive to vinblastine administration.

The two phase-nonspecific agents, doxorubicin and cisplatin, while showing less efficacy in the TS^Q^, maintained a significant cell kill. Cisplatin forms covalent adducts with DNA, the most prevalent being the 1, 2-intrastrand crosslink (Zamble and Lippard, 1995). The DNA adducts are detected by a range of damage recognition proteins, which leads to apoptosis or DNA repair and survival (Siddik, 2003). The relative sensitivities of P- and Q-cells to the initial DNA–platinum adduct formation remains unclear. However, replication is required for the formation of cisplatin-induced DNA double-strand breaks (Kaina, 2003). In addition, Q-cells in solid tumours *in vivo* have previously been shown to have greater DNA repair capacities than the total cell population in response to cisplatin (Masunaga *et al*, 1999). Cell cycle arrest is often considered inhibitory to the induction of cytotoxicity and is known to be important in enabling the nucleotide excision repair machinery to remove cisplatin adducts (Siddik, 2003). Therefore, the decreased sensitivity of the TS^Q^ to cisplatin may be a result of the Q-cells having a greater capacity and/or more time to repair the cisplatin-induced DNA damage.

Doxorubicin displayed a decreased potency in the TS^Q^ compared to the TS^P^, indicating that the cytotoxic effects are only partly proliferation-dependent. A previous study also observed an increase in long-term survival and proliferation potential in doxorubicin-treated Q-cells over P-cells (Siu *et al*, 1999). The proliferation-independent cell death observed in the TS^Q^ could result from one or more of the multiple activities of doxorubicin. These include free radical generation, lipid peroxidation, DNA adduct formation, interference with DNA unwinding and membrane-mediated effects (Gewirtz, 1999; Minotti *et al*, 2004).

To overcome the problem of these chemoresistant quiescent tumour cells, a greater understanding of the mechanisms regulating proliferation in cancer cells and how these operate in a multicellular tumour environment is required. One possible approach to restore chemotherapeutic sensitivity may be to inhibit temporarily the activity of p27^kip1^ and therefore force the Q-cells into the cycle prior to drug administration. The validity of this approach has already been demonstrated *in vitro* by pretreating TS with antisense oligonucleotides to p27^kip1^. This resulted in increased cellular proliferation and sensitised the tumour cells to 4-hydroperoxycyclophosphomide (St Croix *et al*, 1996). Any approach should ideally be selective for the killing of tumour Q-cells over the normal cells of the body, which are also mostly quiescent.

In summary, we have described a new derivative of the MCTS model that mimics quiescent microregions within solid tumours. Like those solid tumours in the clinic with low proliferative fractions, these TS^Q^ show considerable resistance to a panel of established chemotherapeutic agents. The model described here would not only be useful for initial drug screening but also for assessing the long-term efficacy of drugs in quiescent tumour microregions. For example, after exposure to a chemotherapeutic drug, the TS^Q^ could be maintained in a quiescent state for a defined period before being selectively returned to a proliferating state. This would mimic the recruitment of Q-cells to the proliferating fraction that occurs in solid tumours *in vivo*. Using this approach will enable examination of the longevity of drug damage in quiescent microregions and the factors influencing this such as DNA repair capacity. This model may also be useful in evaluating the penetration of viral/non-viral vectors in quiescent tumour tissue and the subsequent protein expression.

## Figures and Tables

**Table 1:** Efficacy of chemotherapeutic drugs in TS

	**TS^P^**		**TS^Q^**	
**Drug**	**IC_50_ (*μ*M)**	**Extent of cell kill (% of total cells)**	**IC_50_ (*μ*M)**	**Extent of cell kill (% of total cells)**
Vinblastine	1.4±0.7	88±5	No IC_50_ at max conc.	34±12
Doxorubicin	0.8±0.2	93±5	13±5^a^	81±10
Cisplatin	3.3±1.2	100±0	84±30^a^	80±12
5-FU	25±8	88±4	No IC_50_ at max conc.	11±11

TS^P^=proliferating tumour spheroids; TS^Q^=quiescent tumour spheroids; 5-FU=5-fluorouracil.

TS^P^ and TS^Q^ were exposed to a range of concentrations of chemotherapeutic drugs (1 nM to 316 *μ*m (up to 1 mM for 5-FU)) for 16 h. The TS were incubated in drug-free medium for a further 6-day (recovery) period to allow the cytotoxic effects of the drugs to be realised. After this time, the methylene blue assay was performed. The potency of the drug to elicit cytotoxicity was expressed as an IC_50_ value determined from dose–response curves as described in the Materials and Methods. The extent of the cell kill in the TS was expressed as a percentage of control for the highest concentration of drug tested. Six replicates were performed for each drug concentration with the values obtained from three or more independent observations being shown as mean±s.e.m.

a Indicates statistical significance (*P*<0.05).
